# Vitamin D status, including serum levels and sun exposure are associated or correlated with bone mass measurements diagnosis, and bone density of the spine

**DOI:** 10.1186/s40795-023-00707-y

**Published:** 2023-03-14

**Authors:** Adeleh khodabakhshi, Sayed Hossein Davoodi, Farhad Vahid

**Affiliations:** 1 Department of Nutrition, Faculty of Public Health, Kerman University of Medical Sciences, Kerman, Iran; 2 Physiology Research Center, Kerman University of Medical Sciences, Kerman, Iran; 3 Department of Cellular and Molecular Nutrition, National Nutrition and Food Technology Research Institute, Faculty of Nutrition Science and Food Technology, Shahid Beheshti University of Medical Sciences, Tehran, Iran; 4 Nutrition and Health Research Group, Department of Precision Health, Luxembourg Institute of Health, Strassen, Luxembourg; 5 Nutrition and Health Research Group, School of Health, Arak University of Medical Sciences, Arak, Iran

**Keywords:** Bone mineral density, Osteoporosis, Osteopenia, Metabolic bone diseases

## Abstract

**Background:**

Osteoporosis is a health complication worldwide, especially in developing countries. The prevalence was reported to be 18.3% globally. While the effect of biochemical factors on fracture risk/odds has been documented, the association/correlation between serum 25(OH)D levels, vitamin D dietary intake, and sun exposure with bone mineral density (BMD) remains controversial. This study aimed to evaluate the association and correlation between vitamin D status, including serum levels, dietary intakes, and sun exposure with BMD. We hypothesized that vitamin D-related factors would have different correlations/associations with BMD, which would help better evaluate future studies’ results.

**Methods:**

A total of 186 individuals were included in this study (winter 2020). BMD was measured by Dual-energy X-ray absorptiometry. Blood serum levels of 25(OH)D, phosphorus, calcium, parathyroid hormone (PTH), and calcitonin were tested using standard lab tests. Valid and reliable questionnaires were used for sun exposure assessment and dietary intakes.

**Results:**

There was a significant protective association between spine BMD (classifications, two groups) (OR = 0.69, 95%CI: 0.50–0.94; p-value = 0.023), BMD diagnosis (classifications, two groups) (OR = 0. 69, 95%CI: 0.49–0.87; p-value = 0.036) and sun exposure. There was a significant and moderate correlation between Spine measurements (Spine BMD: Pearson correlation coefficient = 0.302, p-value = 0.046; Spine T-score: Pearson correlation coefficient = 0.322, p-value = 0.033, Spine Z-score: Pearson correlation coefficient = 0.328, p-value = 0.030) and serum 25(OH)D. In addition, participants with osteopenia and osteoporosis significantly consume a higher amount of soluble fiber than the normal BMD group. There was no significant correlation between vitamin D intake and BMD.

**Conclusion:**

In conclusion, serum 25(OH)D levels and sun exposure are correlated and associated with BMD. However, prospective studies are needed to investigate the association between dietary vitamin D intake and BMD.

## Background

As a systemic disease, osteoporosis is characterized by microarchitectural deterioration of bone tissue and low bone mass [1]. It is a crucial public health problem worldwide, especially in developing countries, so the prevalence of osteoporosis globally was reported to be 18.3% [2]. According to estimates in Iran, about 17% of the general population over 30 years have osteoporosis, and about 35% suffer from osteopenia [3]. If identified early in its course, as it is a major leading cause of bone fragility fractures, many of the fractures can be prevented [4]. Dietary and lifestyle-related factors such as calcium and/or vitamin D deficiency, little or no exercise (sedentary lifestyle), especially weight-bearing exercise, alcohol abuse, smoking, genetic factors, and environmental and hormonal factors, among others, affect bone mineral density (BMD) [5, 6].

While the effect of biomarkers on fracture risk/odds has been documented in some previous studies, the association/correlation between serum 25(OH)D levels, dietary intake, and sun exposure with BMD remains controversial [7, 8]. Although a positive association between low serum vitamin D and low BMD was found in several studies [9–11], other studies did not show any significant association between these two parameters [7, 12, 13].

Until recently, in some countries, such as the UK, vitamin D and/or calcium supplementations were the first treatment choice for preventing/controlling fractures in the elderly [14]. However, the Randomised Evaluation of Calcium Or vitamin D (RECORD) trial questioned/criticized the importance of vitamin D, and apparently, this strategy may not be sufficient to avert further fractures in the ‘healthy’ elderly [15]. Some other randomized controlled trials also were not able to show an advantage in fracture reduction with vitamin D supplementation [16, 17]. However, a meta-analysis of randomized controlled trials proposed that 20 µg/day (800 IU/day) of vitamin D is necessary to demonstrate any advantage [18].

Nevertheless, low vitamin D levels is associated/correlated with higher odds/risk of bone loss, bone turnover, and other bone-related disorders [19]. On the other hand, it seems diet attenuates the seasonal variation of vitamin D levels at the northern latitude, where the quality of sunlight for vitamin D production decreases [19]. Therefore, it might be a comprehensive and advantageous solution to consider all the factors involved in vitamin D status, including exposure to sunlight, dietary intake (with or without supplementation), and serum vitamin D levels to assess its effect on bone health or even other vitamin-related diseases.

Considering that, this study aimed to evaluate the association and correlation between vitamin D status, including serum levels, dietary intakes, and sun exposure with BMD.

## Methods

### Study population

Protocol and design of study previously published elsewhere [8]. Briefly, this study was conducted on 186 Sirjan Gol Gohar Company staff in the winter of 2020. An invitation letter was circulated to all staff, inviting them to participate in the study. Then, individuals who accepted the invitation (responded to the initial letter) and had the inclusion criteria (see below) were included in the survey [8]. Written informed consent was obtained from all participants. The study protocol and design were approved by the Kerman University of Medical Sciences ethics committee board (IR.KMU.REC.1399.156). All methods were performed in accordance with the Declaration of Helsinki.A trained professional filled out a general questionnaire for all participants, including general characteristics and medical history.

### Inclusion and exclusion criteria

Individuals with pregnancy and lactation, diseases interfering with vitamin D absorption/metabolisms such as chronic pancreatitis, inflammatory bowel disease (IBD), resection of part of the intestine or stomach, as well as individuals with hyperparathyroidism, renal failure, advanced liver failure, rheumatoid arthritis, and those who took calcium supplements at least once a day and vitamin D supplements over the past two weeks, and vitamin D ampules over the past six months, individuals smoking more than 10 cigarettes/day and consuming alcohol for more than 5 years and more than a glass/day or individuals with addiction to any drugs were excluded from the study [8].

### Blood samples

In a fasting state, seven milliliters (ml) of blood were taken from the individuals. Blood samples were immediately centrifuged and stored at -80^ °C^. The ELISA method used a Monobind kit made in the USA to measure serum 25(OH)D. In addition, serum calcium and phosphorus were measured using an Auto Analyser (Hitachi, Germany) photometry method. Serum PTH and calcitonin were measured by the Chemiluminescence method (Siemens kit, Germany).

### Dietary intake

Participants’ dietary intakes were estimated by semi-quantitative and valid Food Frequency Questionnaires (FFQ) [20]. A nutritionist completed the questionnaire. Portion size in FFQ was converted to grams per day using household measures. Subsequently, the Nutritionist IV software was applied to extract macro and micronutrients daily intake, including vitamin D [8].

### Sun exposure

Using a valid and reliable questionnaire, sun exposure was estimated. The questionnaire included questions about the amount of exposure to outdoor sunlight (on weekdays and weekends), applying sunscreen creams, and the parts of the body exposed to sunlight during outdoor sunlight [21, 22].

### BMD

An experienced and trained technician assessed hip, femoral neck, and lumbar spine (L1–4) areal BMD g/cm^2^ by Dual-energy x-ray absorptiometry (Hologic Horizon WI, USA). According to the World Health Organization (WHO) classification system, osteoporosis was classified as T-score ≤ − 2.5, osteopenia as − 2.5 < T-score < − 1, and normal as T-score ≥ − 1 [23].

## Statistical analyses

Before choosing statistical tests, the normality of continuous variables was checked by the Q-Q plot and Kolmogorov-Smirnov test. If the variables were not normal, they were log-transformed. An Independent sample t-test was used for continuous variables, and chi-square analyses were used for categorical variables. Bivariate correlation (variables categorized), Spearman’s rho, was used to investigate the correlation between classified/categorized variables. Partial correlation controlled for BMI, age, PTH, and calcitonin was applied to investigate the correlation between two continuous variables while taking away the effects of another variable, or several other variables, on these correlations. Logistic regression models adjusted for age, BMI, PTH, and Calcitonin were used to investigate the association between vitamin status, dietary intake, serum levels, and sun exposure with BMD measurements including spine, total hip, and femoral neck and BMD diagnosis. Data were analyzed with SPSS (IBM, Chicago, IL, USA) version 25.0. A p-value of < 0.05 (2-sided) was considered statistically significant. Benjamini–Hochberg correction was applied to all p-values, and all p-values are displayed after this correction.

## Results

### Distribution of basic characteristics and their comparison

The distribution of anthropometric, socioeconomic, and serum indicators of participants is shown in Table 1. Based on Table 1, there was no significant difference between the normal BMD group and participants with osteopenia and the osteoporosis group in terms of baseline measurements. A comparison of participants’ macro-and micronutrient daily intake is represented in Table 2. According to Table 2, except for soluble fiber (normal BMD group 0.16 ± 0.09 vs. osteopenia and osteoporosis group 0.26 ± 0.18), there was no significant in terms of dietary intakes in the two groups. In addition, Table 2 shows that participants with osteopenia and osteoporosis consume significantly higher amounts of soluble fiber than the normal BMD group.

**Table 1:** Distribution of anthropometric, socioeconomic, and serum indicators of participants

	Mean ± SD or N (%)			P-value*
	Normal	Osteopenia and Osteoporosis	Total	
Age (year)	34.6 ± 9.2	36.6 ± 6.3	35.9 ± 7.7	0.320
BMI (Kg/m^2^)	26.2 ± 3.2	24.9 ± 3.2	26.1 ± 3.7	0.131
PTH (pg/mL)	43.1 ± 19.1	41.5 ± 22.3	44.3 ± 21.4	0.770
Serum calcium (mg/dL)	9.6 ± 0.4	9.74 ± 0.5	9.76 ± 0.5	0.418
Serum phosphorus (mg/dL)	3.1 ± 0.5	3.1 ± 0.3	3.2 ± 0.4	0.841
Calcitonin (pg/mL)	5.6 ± 2.6	6.0 ± 3.8	5.2 ± 2.8	0.663
Serum D3 (ng/mL)	27.4 ± 13.4	27.09 ± 15.4	26.4 ± 13.5	0.922
Gender-Men	25 (96.2%)	36 (97.3%)	61 (96.8%)	0.659
Smoking-No	18 (69.2%)	28 (75.7%)	49 (77.7%)	0.361
Marital status-Married	17 (65.4%)	30 (81.1%)	47 (74.6%)	0.377
Education-University degree	25 (96.2%)	36 (97.3%)	61 (96.8%)	0.419
Exposure to sunlight				0.057
■ ˃30 min	0	7 (18.9%)	7 (18.9%)	
■ 31–60 min	0	3 (8.1%)	3 (8.1%)	
■ 2 h	2 (7.7%)	4 (10.8%)	6 (18.5%)	
■ 3 h	3 (11.5%)	3 (8.1%)	6 (19.6%)	
■ 4 h	4 (15.4%)	5 (13.5%)	9 (28.9%)	
■ 5 h	3 (11.5%)	0	3 (11.5%)	
■ 6 h	4 (15.4%)	7 (18.9%)	11 (34.3%)	

* Independent sample t-test was used for comparing continuous variables. Chi-square analyses were used for comparing categorical variables.

BMI = body mass index, PTH = parathyroid hormone.

Benjamini–Hochberg correction was applied to all p-values: all p-values are displayed after this correction.

**Table 2:** Comparison of participants’ macro-and micronutrient daily intake

	Mean ± SD			P-value*
	Normal	Osteopenia and Osteoporosis	Total	
Total energy (kcal)	1540 ± 629.8	1680 ± 691.4	1617 ± 569.8	0.511
Total protein (g)	64.2 ± 28.1	67.8 ± 25.2	66.2 ± 26.2	0.674
Total carbohydrate (g)	198.1 ± 95.0	236.5 ± 136.4	219.2 ± 119.7	0.319
Total fat (g)	56.1 ± 26.9	53.2 ± 26.2	54.5 ± 26.2	0.737
Cholesterol (mg)	379.3 ± 448.7	314.3 ± 260.5	343.6 ± 345.1	0.570
SFA (g)	17.1 ± 6.4	18.1 ± 9.2	17.6 ± 8.0	0.685
MUFA (g)	19.1 ± 10.2	18.5 ± 11.1	18.8 ± 10.6	0.860
PUFA (g)	13.4 ± 8.5	10.9 ± 4.2	12.1 ± 6.5	0.234
MFA (g)	17.6 ± 9.9	17.0 ± 10.1	17.3 ± 9.9	0.840
PFA2 (g)	11.3 ± 8.2	9.3 ± 4.1	10.2 ± 6.3	0.325
PFA3 (g)	0.87 ± 0.30	0.76 ± 0.52	0.81 ± 0.44	0.457
PFA5 (g)	0.16 ± 0.16	0.09 ± 0.8	0.12 ± 0.13	0.089
PFA6(g)	3.9 ± 5.2	3.0 ± 2.1	3.4 ± 3.8	0.424
Sodium (mg)	1355 ± 671.9	1415 ± 898.1	1388 ± 795.0	0.816
Potassium (mg)	2047 ± 840.1	2416 ± 887.7	2250 ± 875.6	0.188
Vitamin A (RAE)	306.4 ± 184.0	348.5 ± 219.7	329.6 ± 203.0	0.522
Beta-carotene (µg)	690.2 ± 401.7	845.5 ± 502.9	775.6 ± 461.2	0.295
Alpha-carotene (µg)	38.1 ± 26.4	63.5 ± 53.3	52.1 ± 44.7	0.072
Lutein (µg)	829.2 ± 475.1	906.7 ± 520.6	871.8 ± 495.9	0.629
Betacryptox (µg)	201.1 ± 139.9	321.9 ± 265.7	267.5 ± 224.2	0.090
Vitamin C (mg)	57.6 ± 30.7	80.8 ± 51.0	70.4 ± 44.1	0.099
Calcium (mg)	674.6 ± 233.3	776.2 ± 381.1	730.5 ± 323.3	0.329
Iron (mg)	11.1 ± 4.8	12.7 ± 5.7	11.9 ± 5.3	0.326
Vitamin D (µg)	1.8 ± 1.6	2.1 ± 2.7	1.9 ± 2.2	0.707
Vitamin E (mg)	8.7 ± 8.3	6.9 ± 3.6	7.7 ± 6.2	0.372
Alpha-tocopherol (mg)	5.5 ± 5.5	4.4 ± 2.5	4.9 ± 4.1	0.403
Thiamine (mg)	1.49 ± 0.67	1.71 ± 0.99	1.61 ± 0.86	0.441
Riboflavin (mg)	1.47 ± 0.75	1.53 ± 0.68	1.50 ± 0.71	0.790
Niacin (mg)	14.0 ± 6.3	16.0 ± 7.8	15.1 ± 7.1	0.389
Vitamin B6 (mg)	1.11 ± 0.34	1.28 ± 0.58	1.21 ± 0.49	0.277
Total folate (µg)	413.1 ± 184.5	490.7 ± 288.0	455.7 ± 247.1	0.329
Folate DFE (µg)	510.5 ± 269.9	615.5 ± 455.3	568.3 ± 382.3	0.395
Vitamin B12 (µg)	3.89 ± 1.71	4.86 ± 2.77	4.42 ± 2.38	0.203
Biotin (µg)	25.1 ± 23.3	22.9 ± 14.0	23.9 ± 18.5	0.719
Pantothenic acid (mg)	4.6 ± 2.1	5.1 ± 2.6	4.8 ± 2.3	0.540
Vitamin K (µg)	86.1 ± 57.0	97.0 ± 69.8	92.1 ± 63.8	0.596
Phosphorous (mg)	1124 ± 412.6	1209 ± 440.1	1170 ± 424.6	0.537
Magnesium (mg)	268.4 ± 131.3	294.8 ± 108.9	282.9 ± 118.6	0.490
Zinc (mg)	10.8 ± 5.4	12.6 ± 5.0	11.8 ± 5.2	0.301
Copper (mg)	1.03 ± 0.44	1.18 ± 0.46	1.11 ± 0.45	0.321
Manganese (mg)	3.5 ± 1.2	4.2 ± 3.3	3.9 ± 2.6	0.372
Selenium (µg)	102.1 ± 56.1	106.7 ± 50.4	104.6 ± 52.4	0.783
Total fiber (gr)	14.1 ± 5.3	16.5 ± 6.5	15.4 ± 6.0	0.219
Soluble fiber (gr)	0.16 ± 0.09	0.26 ± 0.18	0.22 ± 0.15	**0.043**
Insoluble fiber (gr)	1.12 ± 0.51	1.44 ± 0.73	1.30 ± 0.66	0.120
Crude fiber (gr)	12.2 ± 5.4	13.9 ± 13.3	13.2 ± 10.4	0.615
Total sugar (gr)	49.7 ± 22.0	58.8 ± 23.1	54.5 ± 22.8	0.228
Caffeine (mg)	60.8 ± 51.1	98.2 ± 105.5	81.4 ± 86.5	0.177

*Independent sample t-test was used for comparing continuous variables.

*Benjamini–Hochberg correction was applied to all p-values: all p-values are displayed after this correction; significant values are given in **bold**.

SFA = saturated fatty acid, MUFA = monounsaturated fatty acid, PUFA = polyunsaturated fatty acid, PFA = Polyunsaturated fatty acid,

### Correlations

Partial and bivariate correlations between serum 25(OH)D and BMD are shown in Table 3. According to Table 3, in the partial correlation model controlled for BMI, age, PTH, and calcitonin, there is a significant and moderate correlation between Spine measurements (Spine BMD: Pearson correlation coefficient = 0.302, p-value = 0.046; Spine T-score: Pearson correlation coefficient = 0.322, p-value = 0.033, Spine Z-score: Pearson correlation coefficient = 0.328, p-value = 0.030) and serum 25(OH)D. The partial and bivariate correlation between vitamin D intake and BMD are shown in Table 4. According to Table 4, there was no significant correlation between vitamin D intake and BMD. Partial and bivariate correlations between sun exposure and BMD are shown in Table 5. Table 5 shows that only in bivariate models (BMD are classifications, two groups) without controlling for any confounder factor, there is a significant, moderate, and negative correlation between Spine BMD (correlation coefficient=-0.355, p-value = 0.017), BMD diagnosis (correlation coefficient=-0.326, p-value = 0.029) and sun exposure (Table 5).

**Table 3:** Partial and bivariate correlation between serum 25-hydroxyvitamin D3 and bone mass measurements (BMD).

Variables	Model A		Model B	
	Correlation	P-Value*	Correlation	P-Value*
Spine BMD	**0.302**	**0.046**	-0.119	0.353
Femoral neck BMD	0.029	0.850	0.096	0.456
Total hip BMD	0.033	0.830	0.148	0.246
BMD diagnosis			-0.013	0.918
Spine T-Score	**0.322**	**0.033**		
Femoral neck T-score	0.064	0.681		
Total hip T-score	0.099	0.521		
Spine Z-score	**0.328**	**0.030**		
Femoral neck Z-score	0.067	0.664		
Total hip Z-score	0.071	0.645		

Mode A: Partial correlation controlled for BMI, Age, PTH, and Calcitonin.

Model B: Bivariate correlation (variables categorized).

BMI = body mass index, PTH = parathyroid hormone.

*Benjamini–Hochberg correction was applied to all p-values: all p-values are displayed after this correction; significant values are given in **bold**.

**Table 4:** Partial and bivariate correlation between vitamin D intake and bone mass measurements (BMD).

Variables	Model A		Model B	
	Correlation	P-Value*	Correlation	P-Value*
Spine BMD	-0.056	0.785	0.220	0.172
Femoral neck BMD	-0.017	0.935	0.054	0.739
Total hip BMD	-0.103	0.617	0.230	0.153
BMD diagnosis			0.192	0.235
Spine T-score	-0.063	0.670		
Femoral neck T-score	-0.009	0.967		
Total hip T-score	-0.111	0.591		
Spine Z-score	-0.049	0.811		
Femoral neck Z-score	-0.012	0.953		
Total hip Z-score	-0.109	0.506		

Mode A: Partial correlation controlled for BMI, Age, PTH, and Calcitonin

Model B: Bivariate correlation (variables categorized).

BMI = body mass index, PTH = parathyroid hormone.

*Benjamini–Hochberg correction was applied to all p-values: all p-values are displayed after this correction.

**Table 5:** Partial and bivariate correlation between sun exposure and bone mass measurements (BMD).

Variables	Model A		Model B	
	Correlation	P-Value*	Correlation	P-Value*
Spine BMD	0.171	0.366	**-0.355**	**0.017**
Femoral neck BMD	0.034	0.859	0.053	0.730
Total hip BMD	-0.002	0.992	0.024	0.875
BMD diagnosis			**-0.326**	**0.029**
Spine T-score	0.174	0.377		
Femoral neck T-score	0.031	0.870		
Total hip T-score	-0.025	0.895		
Spine Z-score	0.176	0.351		
Femoral neck Z-score	0.032	0.876		
Total hip Z-score	0.002	0.990		

Mode A: Partial correlation controlled for BMI, Age, PTH, and Calcitonin

Model B: Bivariate correlation (variables categorized).

BMI = body mass index, PTH = parathyroid hormone.

*Benjamini–Hochberg correction was applied to all p-values: all p-values are displayed after this correction; significant values are given in **bold**.

**Fig. 1:**
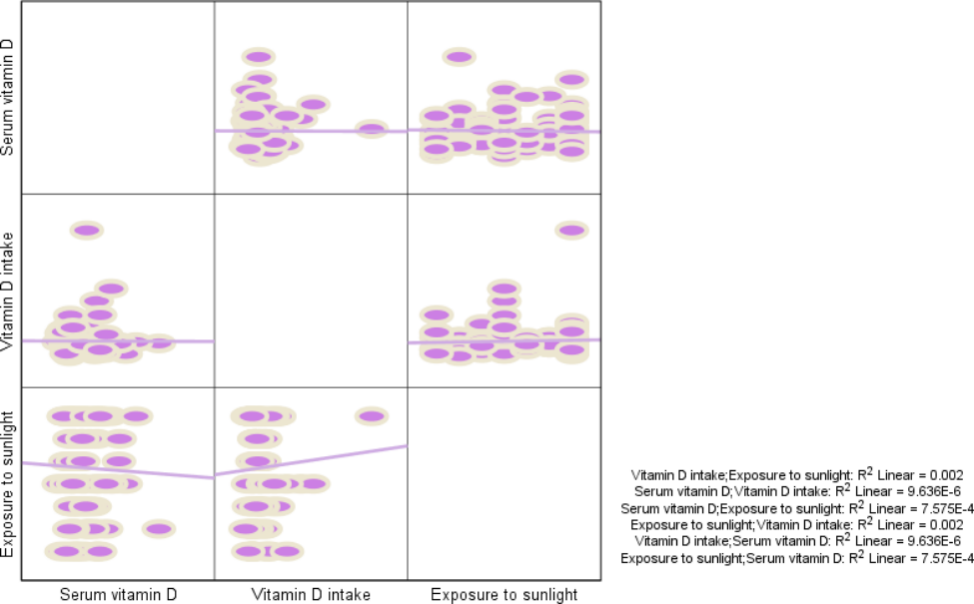
Correlation matrix between vitamin D status, including serum vitamin D, dietary intake, and sunlight exposure

In addition, Fig. 1 represents the correlation matrix between vitamin D status, including serum vitamin D, dietary intake, and sunlight exposure.

### Regression models

Association (OR and 95% CI) between serums 25(OH)D, vitamin D intake, sun exposure, and BMD are shown in Table 6. According to Table 6, in regression logistic multivariable models adjusted for BMI, age, PTH, and calcitonin, there was a significant protective association between spine BMD (classifications, two groups) and serums 25(OH)D (OR = 0.92, 95%CI: 0.86–0.99; p-value = 0.025) and between BMD diagnosis (classifications, two groups) and sun exposure (OR = 0.51, 95%CI: 0.24–0.98; p-value = 0.049). In addition, Table 6 showed that in regression logistic crude models, there was a significant protective association between spine BMD (classifications, two groups) (OR = 0.69, 95%CI: 0.50–0.94; p-value = 0.023) BMD diagnosis (classifications, two groups) (OR = 0. 69, 95%CI: 0.49–0.87; p-value = 0.036) and sun exposure (Table 6). According to Table 6, there was no significant association between vitamin D intake and BMD in regression logistic multivariable and crude models.

**Table 6:** Association (OR95%CI) between serums vitamin D3, vitamin D intake, sun exposure, and bone mass measurements (BMD).

Categories	Serums vitamin D3				Vitamin D intake				Sun exposure			
	Model A		Model B		Model A		Model B		Model A		Model B	
	OR (95%CI)	P-Value*	OR (95%CI)	P-Value*	OR (95%CI)	P-Value*	OR (95%CI)	P-Value*	OR (95%CI)	P-Value*	OR (95%CI)	P-Value*
Spine	0.97 (0.94–1.01)	0.200	**0.92 (0.86–0.99)**	**0.025**	1.10 (0.82–1.49)	0.499	0.68 (0.22–2.10)	0.506	**0.69 (0.50–0.94)**	**0.023**	0.72 (0.46–1.07)	0.111
Total hip	1.02 (0.98–1.06)	0.254	1.00 (0.94–1.05)	0.938	1.22 (0.89–1.67)	0.205	1.01 (0.99–1.04)	0.987	1.02 (0.74–1.41)	0.872	1.24 (0.59-2,63)	0.562
Femoral neck	1.01 (0.97–1.05)	0.452	1.00 (0.95–1.05)	0.833	1.05 (0.79–1.39)	0.733	0.97 (0.29–3.21)	0.967	1.06 (0.80–1.42)	0.662	0.97 (0.64–1.47)	0.905
BMD diagnosis	0.99 (0.96–1.03)	0.921	0.98 (0.93–1.02)	0.417	1.05 (0.78–1.42)	0.701	0. 99 (0.31–3.17)	0.994	**0. 69 (0.49–0.87)**	**0.036**	**0.51 (0.24–0.98)**	**0.049**

Mode A: Crude models

Model B: Models adjusted for age, BMI, PTH, and Calcitonin.

* Logistic regression models.

*Benjamini–Hochberg correction was applied to all p-values: all p-values are displayed after this correction; significant values are given in **bold**.

## Discussion

According to the result of our study, there is a significant and moderate correlation between Spine BMD and serum 25(OH)D. In addition, there is a significant, moderate, and negative correlation between Spine BMD and BMD diagnosis (osteopenia and osteoporosis) with sun exposure. The results of the correlation between serum 25(OH)D levels and BMD values are found to be controversial [8]. While certain studies have failed to find any association between these two variables, others have suggested positive correlations between serum 25(OH)D levels and BMD values.

In line with our finding, Khashayar et al. reported 25(OH)D levels were inversely correlated with BMD values at the total hip and spine in both sexes [24]. In addition, Kamineni concluded Vitamin D deficiency coexists with low BMD [25]. They concluded that vitamin D insufficiency is among the common risk factor for osteoporosis-related to low bone mass and increased bone remodeling [25]. Contrary to these findings, a study on patients with low BMD in the Southeast Asian population concluded that there is no direct association between serum 25(OH)D levels and BMD [26]. Another study revealed no association between BMD and serum vitamin D levels [27].

In addition, Chhantyal et al. reported that free vitamin D was significantly related to lumbar BMD; however, there was no significant association between BMD at different sites as well as fragile vertebral fracture total serum with vitamin D levels [28].

Moreover, our results suggest that sunlight exposure reduced the risk of osteoporosis and osteopenia and increased BMD. This finding aligns with previous studies exploring the links between sunlight exposure and BMD and osteoporosis [29, 30].

Although, in a previous study, we showed a correlation between some factors with vitamin D [31, 32]. Nevertheless, the association between fracture and total vitamin D remains controversial and unclear.

Undoubtedly, osteoporosis is a widely known predisposing factor for fracture, and vitamin D deficiency has been assumed to be a predictor for osteoporotic fractures [33]. Furthermore, vitamin D insufficiency was regarded as an important risk factor for fragile vertebral fractures in women and men [34]. A study of community-dwelling postmenopausal women found that sufficient vitamin D status might decrease—the risk of future fracture risk [35].

Discrepancies and inconsistency between studies may be attributed to (a) many of these population-based studies have recruited subjects with relatively good health status and, therefore, the lower prevalence of severe vitamin D deficiency and osteoporosis; (b) this study’s sites used for densitometry measurement affect the possible association between 25(OH)D and BMD; (c) also, sex, age, and physical activity vary in these studies.

Surprisingly, there was no significant difference between dietary intakes in the two groups in our study. Still, participants with osteopenia and osteoporosis significantly consumed a higher amount of soluble fiber than the normal BMD group. On the contrary, in the Framingham Offspring Study, associations with hip bone loss were not observed for women, although higher dietary fiber intake may modestly lower bone loss in men at the hip [36]. Data about the relation between fiber and bone turnover biomarkers showed either an increase, decrease, or no changes in bone formation and resorption markers [36].

Our study had its strengths included; this is the first study in Iran that considers all factors related to vitamin D status. Given the geographical location and the high prevalence of vitamin D deficiency in Iran, this study can help interpret the situation of vitamin D deficiency in Iran and countries with similar geographical conditions. Studies have shown that measurement methods can partially explain the lack of correlation between factors [36]. Another strength of our research is using standard methods to measure serum vitamin D and diagnose bone problems. In addition, the use of a valid FFQ and its completion by a nutritionist also assured us that the recall bias, one of the most common biases in retrospective studies, has been minimized.

Like any other study, our study had its limitations. One of our study s limitations was sample loss. So that some patients did not go to the BMD measurement center due to the COVID-19 situation (quarantine); since this problem was not anticipated at the time of study design, the COVID-19 pandemic outbreak also affected our sampling, and we lost some participants for the final analysis. To this end, modification in sampling protocols may be necessary for future studies. Therefore, risk management and quality assurance should be done more carefully and revised for future studies. Another limitation of our study was that it was not representative, so regarding variables such as age and gender, our study participants were not representative of the general population. Since this study was only a pilot study and the study population was deliberately selected from Sirjan Gol Gohar Company staff to highlight the job status more. Therefore, future studies with a large sample size and considering age and sex, and other confounding factors are necessary to confirm the results of our study. Another limitation of our study was the high risk of recall bias due to its retrospective nature. However, by taking the help of trained experts to collect data and complete the questionnaires, we were able to minimize this bias to a certain extent. On the other hand, using blood samples and serum levels of indicators allowed us to examine the data more precisely.

## Conclusion

In conclusion, although the results of our study showed a significant association/correlation between some components of vitamin D status, such as exposure to sunlight or serum levels, we failed to demonstrate the association between dietary vitamin D intake and BMD. Nevertheless, our results support previous studies, which concluded that serum 25(OH)D levels and sun exposure are correlated with bone mass. Future prospective studies considering confounding factors are recommended to confirm the results and elucidate possible mechanisms.

## Data Availability

Data described in the manuscript, codebook, and analytic code will be available upon request pending application and approval by the corresponding author.
